# Associations of Secondary Traumatic Stress and Emotional Labor with Suicidal Ideation Among Emergency Dispatch Personnel in South Korea: A Cross-Sectional Moderated Mediation Analysis

**DOI:** 10.3390/healthcare14213612

**Published:** 2026-10-26

**Authors:** Nayoon Lee, Young Hee Kim

**Affiliations:** 1 College of Nursing, Dong-A University, G11-517, 32-7 Mangyang-ro 111, Seo-gu, Busan 49201, Republic of Korea; 095750@dau.ac.kr; 2 Graduate School, College of Nursing, Dong-A University, G11-517, 32-7 Mangyang-ro 111, Seo-gu, Busan 49201, Republic of Korea

**Keywords:** psychological trauma, depression, suicidal ideation, social support, occupational stress

## Abstract

**Highlights:**

**What are the main findings?**

• Secondary traumatic stress (STS) was significantly associated with both depression and suicidal ideation among South Korean 119 dispatch center workers.
• Depression showed the strongest association with suicidal ideation, and a significant conditional indirect effect of STS on suicidal ideation through depression was observed.
• Emotional labor was positively associated with depression in the focal-predictor model but was not independently associated with suicidal ideation after adjustment for STS and depression.

**What are the implications of the main findings?**

• Higher social support was associated with weaker conditional associations in the primary PROCESS analyses, although moderation findings involving suicidal ideation were not robust in the Tobit sensitivity analysis.
• Screening for STS and depression, together with timely referral, may be relevant components of future occupational mental health programs for emergency dispatch center workers.

**Abstract:**

Background: Emergency dispatch personnel are repeatedly exposed to callers’ distress and emotionally demanding interactions, yet suicidal ideation in this workforce remains understudied. This study examined associations of secondary traumatic stress (STS) and emotional labor with suicidal ideation, including indirect associations through depression and conditional associations involving social support. Methods: A cross-sectional correlational study was conducted among 211 fire-service personnel assigned to emergency dispatch centers in Busan and Gyeongnam, South Korea. Data were analyzed using Hayes’ PROCESS macro Model 59 with HC3 standard errors and 5000 bootstrap resamples. Results: Social support moderated the associations of STS with depression and suicidal ideation and the association of depression with suicidal ideation. The conditional indirect association of STS with suicidal ideation through depression decreased across low, mean, and high social support (0.191, 0.076, and 0.013). For emotional labor, the emotional labor × social support interaction was significant in the depression model, and its association with depression and indirect association with suicidal ideation through depression were supported at low and mean, but not high, social support. Conclusions: STS and depression were associated with suicidal ideation, and the indirect association of STS through depression was weaker at higher social support. However, moderation findings involving suicidal ideation should be interpreted cautiously because the corresponding interaction terms were not statistically significant in the Tobit sensitivity analysis.

## 1. Introduction

### 1.1. Background

Firefighters serve on the front lines of disaster scenes—fire suppression, rescue, and emergency medical response—and are repeatedly exposed to direct experiences of death or serious injury as well as indirect trauma through witnessing or hearing about others’ harrowing incidents [1,2]. Such sustained trauma exposure is associated with an elevated risk of a range of mental health problems, including posttraumatic stress disorder (PTSD), depression, and anxiety [3,4,5]. However, prior research has primarily focused on personnel who respond directly to emergency scenes, whereas comparatively less attention has been given to emergency dispatch personnel who receive and coordinate emergency calls.

Emergency dispatch personnel working within South Korea’s 119 emergency response system are responsible for receiving emergency calls, issuing dispatch orders, and supporting on-scene response [6]. Although they are physically separated from emergency scenes, their work involves repeated exposure to distressing narratives, intense emotional demands, and time-critical decision making. Recent surveys have reported substantial mental health concerns among dispatch center workers; in the National Fire Agency’s 2024 Nationwide Mental Health Survey, the proportion screening positive on a suicide-risk indicator was highest among dispatch center personnel compared with fire suppression, EMS, and rescue personnel [7]. These findings indicate a need for focused investigation of psychological factors associated with suicidal ideation among emergency dispatch personnel in South Korea.

The psychological demands of emergency dispatch work may be particularly relevant to secondary traumatic stress (STS). These workers are repeatedly exposed to others’ traumatic experiences through listening to callers’ cries, screams, and vivid accounts of accident situations, which may contribute to STS symptoms [8]. Figley (1995) defined STS as a condition in which PTSD-like symptoms emerge through repeated exposure to another person’s traumatic experience [9], and although dispatch center workers are physically separated from the scene, the nature of their work—reconstructing traumatic situations from auditory information—may be associated with the development of STS symptoms [8,10]. Although STS overlaps symptomatically with PTSD, the two constructs are not equivalent. STS specifically refers to trauma-related symptoms associated with indirect or secondary exposure to others’ traumatic experiences, whereas PTSD is a clinical disorder defined by established diagnostic criteria [11]. STS is also conceptually distinct from burnout and general occupational stress: burnout primarily reflects responses to chronic work-related strain [12], whereas general occupational stress encompasses a broader range of job demands that are not necessarily trauma-related [13]. Thus, STS was examined in the present study as a trauma-specific occupational stress response rather than as a proxy for PTSD, burnout, or general occupational stress. Indeed, studies conducted abroad have reported STS, PTSD symptoms, depression, anxiety, and suicidal ideation among 911 dispatchers and similar occupational groups [14,15,16], suggesting that indirect auditory exposure to traumatic events warrants consideration in this occupational group.

At the same time, these workers face substantial emotional labor demands. Hochschild (1983) defined emotional labor as the process of regulating and suppressing one’s own emotions to conform to the emotional expression norms required by an organization [17]. While required to make accurate judgments and decisions within a limited time, emergency dispatch personnel must maintain a calm and controlled demeanor even while responding to callers who express fear and anger [18,19]. Previous studies have reported associations of emotional labor with negative mental health outcomes such as depression, burnout, and suicidal ideation [20,21,22]. In particular, STS and emotional labor may represent related occupational stressors; previous research has reported an association between the surface acting component of emotional labor and STS [23]. Examining both factors simultaneously may therefore provide a more comprehensive understanding of psychological distress among emergency dispatch personnel.

To understand the statistical associations linking secondary traumatic stress and emotional labor to suicidal ideation, it is also necessary to consider depression as a potential statistical mediator. According to Hobfoll’s (1989) conservation of resources theory, repeated stress experiences deplete an individual’s psychological resources, and negative affective responses such as depression can emerge in the process of this resource loss [24]. From this perspective, repeated indirect trauma exposure and sustained emotional regulation demands may contribute to the depletion of emotional and psychological resources, which may in turn be associated with depressive symptoms. Depressive symptoms are strongly associated with suicidal ideation, providing a theoretically plausible statistical link between occupational stressors and suicidal ideation. Indeed, prior studies of firefighters and other emotional-labor occupations have reported that trauma-related stress and emotional labor are significantly associated with depression, as well as indirect associations with suicidal ideation through depression [25,26,27,28]. Depression may therefore represent a plausible statistical mediator through which STS and emotional labor are associated with suicidal ideation.

Meanwhile, these associations may vary according to the level of social support an individual perceives. Social support refers to perceived emotional support from family, friends, and significant others [29], and according to Cohen and Wills’ (1985) buffering hypothesis, rather than eliminating the stressful event itself, social support may modify the association between stressful experiences and psychological outcomes [30]. Within a conservation-of-resources perspective, social support may also serve as an interpersonal resource that helps individuals preserve or replenish psychological resources when facing occupational stress. Thus, higher perceived social support may weaken the association of STS or emotional labor with depression, attenuate the association of depression with suicidal ideation, or reduce the direct association of occupational stressors with suicidal ideation. Among Korean firefighters, perceived social support has been reported to moderate the association between trauma exposure and PTSD symptoms [31]. Studies conducted abroad have similarly found that, among 911 dispatchers and similar occupational groups, social support is negatively associated with PTSD symptoms and depressive symptoms [32,33], and a study of firefighters reported that support from family and friends moderates the relationship between job stress and suicidal ideation [34]. Taken together, COR theory and the stress-buffering hypothesis provide complementary explanations for examining social support as a moderator across multiple pathways linking occupational stressors, depression, and suicidal ideation.

Despite this evidence, important gaps remain. Research on suicidal ideation among emergency dispatch personnel in South Korea is limited, and few studies have examined STS and emotional labor simultaneously in this population. In addition, limited evidence is available regarding whether depression statistically accounts for indirect associations of these occupational stressors with suicidal ideation and whether social support conditions different components of these associations. Among prior studies, Kim and Ji [25] provided the closest prior model by examining a moderated mediation pathway from PTSD to suicidal ideation through depression with social support as a moderator in Korean firefighters; the present study extends this framework to emergency dispatch personnel by examining STS and emotional labor across multiple conditional pathways.

Based on the theoretical framework and prior evidence reviewed above, the following hypotheses were proposed. First, higher levels of STS and emotional labor were expected to be associated with greater suicidal ideation. Second, STS and emotional labor were expected to show positive indirect associations with suicidal ideation through depression. Third, perceived social support was expected to weaken the positive associations of STS and emotional labor with depression, the positive association of depression with suicidal ideation, and the direct associations of STS and emotional labor with suicidal ideation. Accordingly, the indirect associations of STS and emotional labor with suicidal ideation through depression were expected to be weaker at higher levels of perceived social support.

### 1.2. Purpose

The purpose of this study was to examine the associations of secondary traumatic stress and emotional labor with suicidal ideation among emergency dispatch personnel in South Korea and to evaluate statistical indirect associations through depression and conditional associations involving social support. By clarifying these associations, this study aimed to provide evidence that may inform future occupational mental health monitoring and the development of support strategies for emergency dispatch personnel.

## 2. Materials and Methods

### 2.1. Study Design

**Figure 1:**
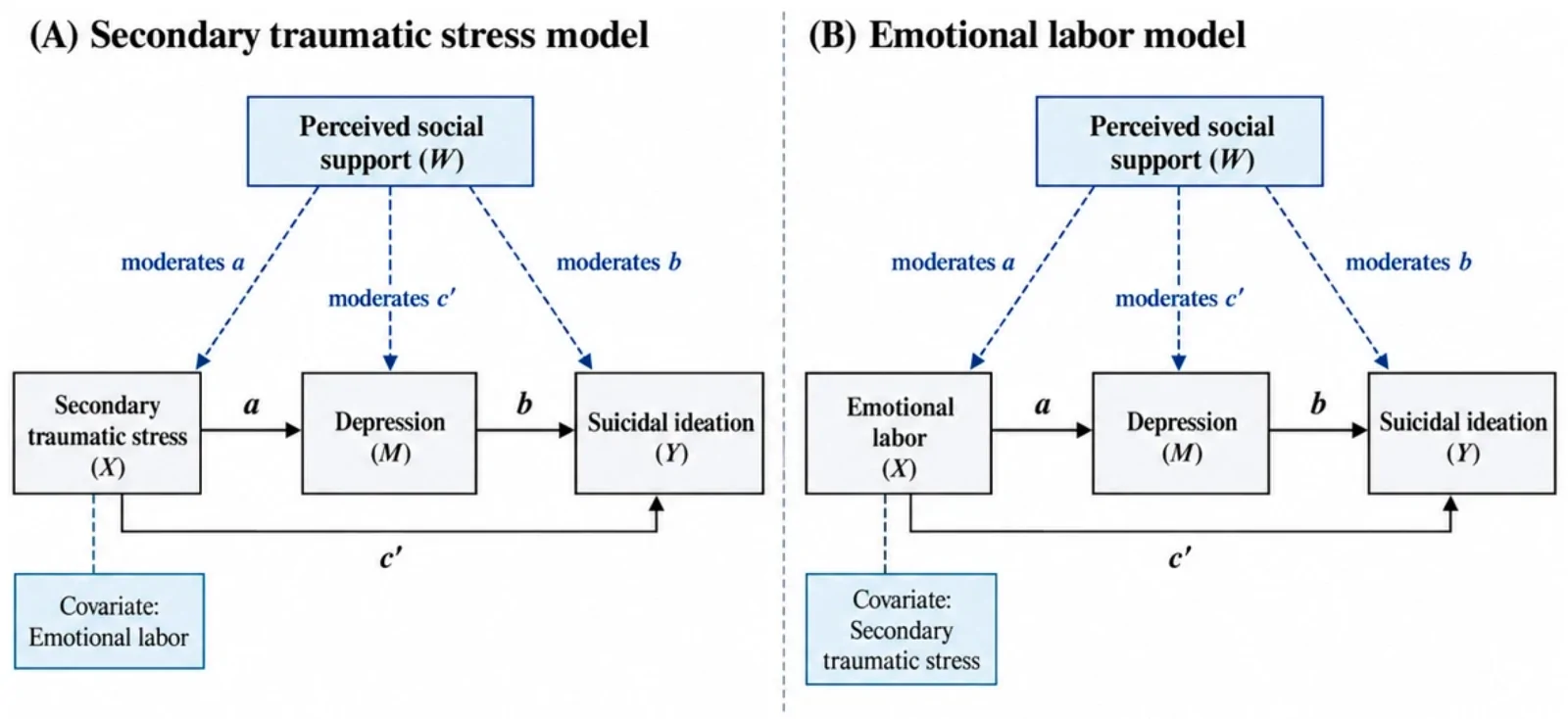
Hypothesized moderated mediation models. Note. Blue dashed arrows from perceived social support indicate moderation of the a, b, and c′ paths, and dashed lines from the covariate boxes indicate covariate adjustment. Secondary traumatic stress and emotional labor were examined as focal predictors in separate models. The alternate occupational stressor was controlled in each model. Additional covariates specified in Section 2.4 are not shown for simplicity.

This study was a descriptive correlational study examining the associations of secondary traumatic stress and emotional labor with suicidal ideation among emergency dispatch personnel, with depression considered as a statistical mediator and perceived social support as a moderator. The hypothesized primary conditional process model is presented in Figure 1. In separate models, secondary traumatic stress and emotional labor were each specified as the focal predictor (X), depression as the statistical mediator (M), suicidal ideation as the outcome (Y), and total perceived social support as the moderator (W). Social support was hypothesized to moderate three associations: the focal predictor–depression path (a path), the depression–suicidal ideation path (b path), and the direct focal predictor–suicidal ideation path (c′ path). The indirect association through depression was represented by the product of the a and b paths. The alternate occupational stressor was included as a covariate in each model.

### 2.2. Participants and Data Collection

This study was conducted with fire service personnel assigned to emergency dispatch centers affiliated with the Busan and Gyeongnam Fire Headquarters. Eligible participants were fire service personnel who had at least six months of work experience in an emergency dispatch center, understood the purpose and content of the study, and voluntarily agreed to participate. Civilian dispatch personnel were not eligible for participation. Individuals who were away from their regular duties because of a leave of absence or training assignment, and those who had difficulty reading and completing the questionnaire, were excluded.

The original sample size was planned using G*Power 3.1 (Heinrich Heine University Düsseldorf, Düsseldorf, Germany) for linear multiple regression (fixed model, R^2^ deviation from zero), assuming a medium effect size (f^2^ = 0.15), a significance level of 0.05, and statistical power of 0.95. Cohen’s conventional medium effect size was used because no previous study provided a stable effect-size estimate for the full conditional process model. At the planning stage, the analytic model included 14 predictor terms (nine covariate terms and five study-variable and interaction terms), for which the minimum required sample size was 194. Allowing for potential dropout, we aimed to recruit a total of 220 participants. After the primary analysis was revised to PROCESS Model 59, sample adequacy was reassessed using the regression equation with the largest number of predictor terms, namely the suicidal ideation outcome equation. This equation included 15 predictor terms: nine covariate terms representing age, dispatch-center work experience, religion, marital status, and rank, and six study-variable and interaction terms comprising the focal predictor, the alternate occupational stressor, depression, social support, the focal predictor × social support interaction, and the depression × social support interaction. Under the same assumptions, the minimum required sample size was 199. The final analytic sample of 211 participants exceeded this requirement.

The researcher visited the emergency dispatch centers affiliated with the Busan and Gyeongnam Fire Headquarters in person to explain the purpose and procedures of the study and to request cooperation with data collection. Recruitment notices containing a QR code were then posted on bulletin boards at the participating centers and circulated through the internal communication network. The survey was administered online through the QR code. Participants first viewed the study information sheet and electronic consent form and could proceed to the questionnaire only after providing consent. Because the exact number of eligible personnel who had an opportunity to view the recruitment notice during the study period could not be determined, a response rate could not be calculated. Personnel who were on leave or otherwise absent during the recruitment period could not be reliably identified for this purpose. A total of 211 participants completed the survey between 17 May and 17 June 2026, and all were included in the final analytic sample. Participants who completed the survey received a small token of appreciation.

All questionnaire items in Google Forms (Google LLC, Mountain View, CA, USA) were set as required, and therefore no item-level data was missing. Google Forms was configured to restrict each respondent to a single submission. No responses were excluded after submission on the basis of response time, straight-lining, or response-pattern inconsistency. Accordingly, all 211 completed questionnaires were included in the final analytic sample.

### 2.3. Measurements

The structured online questionnaire consisted of items on participants’ demographic and occupational characteristics (sex, age, religion, marital status, rank, type of duty, total years of service, and years of experience at the dispatch center), together with instruments measuring secondary traumatic stress (K-STSS), emotional labor (K-ELS), depression (CES-D), suicidal ideation, and social support (MSPSS). All scale scores were calculated by summing item responses. In the present sample, Cronbach’s α was 0.86 or higher for all measures.

#### 2.3.1. Secondary Traumatic Stress

Secondary traumatic stress was measured using the Korean Secondary Traumatic Stress Scale (K-STSS), which was adapted and validated by Park Ji-young (2011) for Korean trauma-related helping professionals [35] from the Secondary Traumatic Stress Scale (STSS) originally developed by Bride, Robinson, Yegidis, and Figley (2004) [36]. The K-STSS consists of 13 items across two subdomains: 9 intrusion items and 4 avoidance items. Each item is rated on a 5-point Likert scale (1 = not at all, 5 = very much so), with higher scores indicating a higher level of secondary traumatic stress. In Park’s (2011) study [35], Cronbach’s α for the overall scale was 0.90 or higher, and in this study Cronbach’s α for the overall scale was 0.953.

#### 2.3.2. Emotional Labor

Emotional labor was measured using the Korean Emotional Labor Scale (K-ELS) developed by Jang Se-jin et al. (2014) [37]. This instrument consists of 24 items across five subdomains: emotion regulation demands and requirements (5 items), overload and conflict in customer response (3 items), emotional dissonance and injury (6 items), organizational monitoring and surveillance (3 items), and organizational support and protection system (7 items). Each item is rated on a 4-point Likert scale (1 = not at all, 4 = very much so), and the organizational support and protection system subdomain is reverse-scored, such that higher scores on this subdomain indicate a greater lack of organizational support and protection. Higher total scores indicate a higher level of emotional labor. Jang et al. (2014) [37] reported subdomain-level Cronbach’s α values ranging from 0.76 to 0.90, and in this study Cronbach’s α for the overall scale was 0.891.

#### 2.3.3. Depression

Depression was measured using the Integrated Korean version of the Center for Epidemiologic Studies Depression Scale (CES-D), which was integrated and validated for the Korean context by Chon Kyum-koo, Choi Sang-jin, and Yang Byung-chang (2001) [38], based on the original CES-D developed by Radloff (1977) [39]. This instrument consists of 20 items across four subdomains: somatic and depressed affect (10 items), emotional distress (5 items), interpersonal relations (2 items), and positive affect (3 items), and measures the frequency of depressive symptoms experienced during the past week. Each item is rated on a 4-point Likert scale, from 0 = rarely or none of the time (less than 1 day) to 3 = most or all of the time (5–7 days). The three items measuring positive affect are reverse-scored. Higher scores indicate a higher level of depression. Chon et al. (2001) [38] reported Cronbach’s α of 0.91, and Cronbach’s α was 0.869 in the present sample.

#### 2.3.4. Suicidal Ideation

Suicidal ideation was measured using six items that were revised and supplemented for the 13th wave of the Korea Welfare Panel Study [40], based on the Suicidal Ideation Questionnaire (SIQ) originally developed by Reynolds (1988) [41]. These items assessed the frequency of suicidal ideation over the past month, with each item rated on a 7-point Likert scale (0 = never, 6 = almost every day). Higher scores indicate a higher level of suicidal ideation. A previous study using the same six-item measure reported that exploratory factor analysis supported a single-factor structure and demonstrated high internal consistency (Cronbach’s α = 0.94) [42]. In the present study, Cronbach’s α was 0.972. Because the previous psychometric evidence was obtained from an adolescent sample, further validation in adult occupational populations is warranted.

#### 2.3.5. Social Support

Social support was measured using the Multidimensional Scale of Perceived Social Support (MSPSS), developed by Zimet, Dahlem, Zimet, and Farley (1988) [29] and adapted into Korean by Shin Jun-sup and Lee Young-boon (1999) [43]. This study applied the Korean-language, 5-point version of the MSPSS as adapted by Shin and Lee. This instrument consists of 12 items across three subdomains: family support (4 items), friend support (4 items), and significant other support (4 items). Each item is rated on a 5-point Likert scale (1 = not at all, 5 = very much so), with higher scores indicating a higher level of perceived social support. The total MSPSS score was used as the moderator in the primary analyses. Zimet et al. (1988) [29] reported Cronbach’s α of 0.85, and Shin and Lee (1999) [43] reported Cronbach’s α of 0.89; in the present study, Cronbach’s α for the overall scale was 0.968.

### 2.4. Data Analysis

The collected data were analyzed using IBM SPSS Statistics version 29.0 (IBM Corp., Armonk, NY, USA) and Hayes’ PROCESS macro version 5.0 for SPSS (Andrew F. Hayes, University of Calgary, Calgary, AB, Canada) [44]. Participants’ general characteristics were presented as frequencies and percentages, and the main study variables—secondary traumatic stress, emotional labor, depression, suicidal ideation, and social support—were analyzed descriptively using means and standard deviations. Differences in the main variables according to participant characteristics were examined using independent-samples *t*-tests or one-way ANOVA. Assumptions relevant to these comparisons, including homogeneity of variance, were examined before interpretation. When the homogeneity-of-variance assumption was not satisfied, Welch’s *t*-test or Welch’s ANOVA was used, as appropriate. Significant conventional one-way ANOVAs were followed by Scheffé post hoc comparisons, whereas significant Welch ANOVAs were followed by Games–Howell tests.

The primary analyses followed the conceptual model shown in Figure 1. Conditional process analyses were conducted using Hayes’ PROCESS macro Model 59, with secondary traumatic stress and emotional labor examined in separate models as the focal predictor. Model 59 allows social support to moderate the association between the focal predictor and depression (a path), the association between depression and suicidal ideation (b path), and the direct association between the focal predictor and suicidal ideation (c′ path) within the same model. In each model, the alternate occupational stressor was included as a covariate. The total social support score was used in the primary Model 59 analyses.

To address the risk of Type I error arising from multiple testing, analyses were distinguished between primary hypothesis-testing analyses and descriptive or exploratory analyses. The PROCESS Model 59 analyses using the total social support score constituted the primary analyses, whereas bivariate correlations and group comparisons across participant characteristics were treated as descriptive or exploratory analyses. No formal multiplicity adjustment was applied to the exploratory inferential analyses. Results from the exploratory group comparisons were therefore interpreted cautiously and regarded as hypothesis-generating rather than confirmatory.

In the regression models that included interaction terms, the focal predictor, depression, and social support were mean-centered to reduce non-essential multicollinearity between interaction terms and their component variables and to facilitate interpretation. Conditional effects were evaluated at the mean and at one standard deviation below and above the mean of social support. Statistically supported interaction effects were further examined using the Johnson–Neyman technique to identify regions of significance. Because Model 59 specifies moderation of both the a and b paths, moderated mediation was evaluated using conditional indirect effects at specified levels of social support rather than a single linear index of moderated mediation. In all moderated mediation analyses, 95% bootstrap confidence intervals were estimated using 5000 bootstrap resamples, and conditional indirect effects were considered statistically supported when the confidence interval did not include zero.

Regression diagnostics were examined before interpretation of the conditional process models. Residual distributions did not indicate substantial departures from normality, and no statistically significant outlier was identified after Bonferroni correction. Because the Breusch–Pagan test indicated heteroskedasticity, heteroskedasticity-consistent HC3 standard errors were used. Independence of observations was supported by the study design, as each participant contributed a single response. Given the substantial proportion of zero scores for suicidal ideation, a Tobit model was additionally estimated as a sensitivity analysis to assess the robustness of the findings to the zero-heavy outcome distribution.

Covariates were selected based on their potential relevance to occupational exposure and suicidal ideation, together with their associations with the study outcomes in the present sample. Age, rank, and dispatch center work experience were considered relevant markers of career stage and occupational exposure, whereas religion and marital status were retained because they were associated with suicidal ideation in the preliminary analyses and represent psychosocial characteristics potentially related to the outcome. In each focal-predictor model, the alternate occupational stressor was additionally included as a covariate. Total career duration was considered but was not simultaneously included because of multicollinearity with other career-related covariates. Sex and duty type were also considered as potential covariates but were not retained in the primary models because neither showed significant associations with depression or suicidal ideation in the preliminary group comparisons.

Given the cross-sectional design, the terms “mediation” and “moderation” are used in a statistical sense throughout this paper; indirect and conditional effects describe patterns of association and should not be interpreted as evidence of causal or temporal pathways.

For the primary conditional process analyses, unstandardized regression coefficients (B), HC3 standard errors, 95% confidence intervals, t statistics, and *p* values were reported. Model-level explanatory power was summarized using R^2^ and adjusted R^2^, and incremental effect sizes for interaction terms were reported as changes in R^2^ (ΔR^2^). Conditional indirect effects were reported with bootstrap standard errors and 95% bootstrap confidence intervals.

### 2.5. Ethical Considerations

Prior to data collection, this study was approved by the Institutional Review Board (IRB) of Dong-A University (IRB No. 2-1040709-AB-N-01-202604-HR-018-02). Participants provided electronic informed consent after receiving information about the study purpose and procedures, the voluntary nature of participation, their right to discontinue participation without disadvantage, and measures to protect their personal information. Because the survey was anonymous and no identifying information was collected, the research team could not identify or individually follow up with participants who reported high levels of suicidal ideation. In accordance with the IRB’s recommendation regarding possible psychological discomfort during participation, all participants were provided with information on a professional counseling service for Korean fire service personnel, including a 24-h counseling hotline. The collected data were used only for research purposes, were securely managed to prevent personal identification, and will be retained for three years after the completion of the study before being permanently deleted.

## 3. Results

### 3.1. General Characteristics of Participants

Participants comprised 150 men (71.1%) and 61 women (28.9%), with a mean age of 39.37 ± 7.88 years. Of the participants, 64.0% reported no religious affiliation and 68.7% were married; the most common rank was Fire Sergeant. The mean total length of service was 11.56 ± 7.45 years, and the mean length of dispatch center experience was 4.09 ± 3.76 years (Table 1).

Exploratory group comparisons indicated differences in depression and suicidal ideation according to age, religion, marital status, rank, total career duration, and dispatch center work experience. Post hoc comparisons showed that participants aged 30–39 years had higher depression and suicidal ideation scores than both the ≤29-year and ≥40-year groups. Participants without a religious affiliation also had higher depression and suicidal ideation scores than those with a religious affiliation. Unmarried participants had higher depression scores than married participants, whereas unmarried and divorced/separated participants had higher suicidal ideation scores than married participants. Social support also differed by marital status, with divorced/separated participants reporting lower social support than unmarried and married participants. Additional post hoc group differences according to rank, total career duration, and dispatch center work experience are presented in Table 2.

### 3.2. Descriptive Findings and Correlations of the Study Variables

Descriptive statistics and correlations among the main study variables are presented in Table 3. Mean scores of the main variables were as follows: secondary traumatic stress, 29.36 ± 10.63; emotional labor, 66.29 ± 9.81; depression, 16.04 ± 8.97; suicidal ideation, 3.07 ± 5.81; and social support, 45.97 ± 11.44. Using a CES-D screening threshold of 16, 90 participants (42.7%) scored at or above the threshold. The mean CES-D score was 16.04, close to this screening threshold. These CES-D values are descriptive screening indicators and do not represent a clinical diagnosis of depression. Cronbach’s α values for the study measures ranged from 0.869 to 0.972.

Correlation analysis showed that suicidal ideation was significantly and positively correlated with secondary traumatic stress (r = 0.645, *p* < 0.001), emotional labor (r = 0.293, *p* < 0.001), and depression (r = 0.797, *p* < 0.001), and significantly negatively correlated with social support (r = −0.658, *p* < 0.001). Secondary traumatic stress was also significantly and positively correlated with emotional labor (r = 0.552, *p* < 0.001) and depression (r = 0.733, *p* < 0.001). Social support was significantly and negatively correlated with emotional labor (r = −0.289, *p* < 0.001), depression (r = −0.663, *p* < 0.001), and suicidal ideation (r = −0.658, *p* < 0.001). The correlation between depression and suicidal ideation was strong (r = 0.797, *p* < 0.001) (Table 3). Suicidal ideation scores were zero for 138 participants (65.4%), whereas 73 participants (34.6%) reported a score greater than zero. The distribution was right-skewed (skewness = 2.244, kurtosis = 4.680).

### 3.3. Primary Conditional Process Analyses

#### 3.3.1. Secondary Traumatic Stress as the Focal Predictor

In the model with secondary traumatic stress as the focal predictor, the regression equation for depression was statistically significant (R^2^ = 0.729, adjusted R^2^ = 0.711, F(HC3) = 39.302, *p* < 0.001). At the mean level of social support, secondary traumatic stress was positively associated with depression (B = 0.3952, HC3 SE = 0.0463, t = 8.537, *p* < 0.001, 95% CI [0.304, 0.486]), whereas emotional labor, included as the alternate occupational stressor, was not significantly associated with depression (B = 0.0890, HC3 SE = 0.0521, t = 1.710, *p* = 0.089, 95% CI [−0.014, 0.192]). At the mean level of secondary traumatic stress, social support was negatively associated with depression (B = −0.1951, HC3 SE = 0.0405, t = −4.822, *p* < 0.001, 95% CI [−0.275, −0.115]). The interaction between secondary traumatic stress and social support was statistically significant (B = −0.0196, HC3 SE = 0.0030, t = −6.530, *p* < 0.001, 95% CI [−0.026, −0.014], ΔR^2^ = 0.058), indicating that the positive association between secondary traumatic stress and depression was weaker at higher levels of social support. The conditional association remained statistically significant at low, mean, and high levels of social support, with coefficients of 0.620 (95% CI [0.490, 0.749]), 0.395 (95% CI [0.304, 0.486]), and 0.171 (95% CI [0.076, 0.266]), respectively (Table 4).

For suicidal ideation, the overall regression equation was statistically significant (R^2^ = 0.821, adjusted R^2^ = 0.807, F(HC3) = 28.115, *p* < 0.001). At the mean level of social support, secondary traumatic stress (B = 0.1404, HC3 SE = 0.0327, t = 4.292, *p* < 0.001, 95% CI [0.076, 0.205]) and depression (B = 0.1932, HC3 SE = 0.0447, t = 4.319, *p* < 0.001, 95% CI [0.105, 0.281]) were positively associated with suicidal ideation. At the mean levels of secondary traumatic stress and depression, the conditional coefficient for social support was negative but not statistically significant (B = −0.0195, HC3 SE = 0.0285, t = −0.685, *p* = 0.494, 95% CI [−0.076, 0.037]). Both the secondary traumatic stress × social support interaction (B = −0.0084, HC3 SE = 0.0030, t = −2.748, *p* = 0.007, 95% CI [−0.014, −0.002], ΔR^2^ = 0.009) and the depression × social support interaction (B = −0.0101, HC3 SE = 0.0034, t = −2.964, *p* = 0.003, 95% CI [−0.017, −0.003], ΔR^2^ = 0.011) were statistically significant. Emotional labor was not significantly associated with suicidal ideation in this model (B = −0.0015, HC3 SE = 0.0259, t = −0.057, *p* = 0.955, 95% CI [−0.053, 0.050]) (Table 5). Conditional direct and indirect associations across levels of social support are presented in Section 3.4.

#### 3.3.2. Emotional Labor as the Focal Predictor

In the model with emotional labor as the focal predictor, the regression equation for depression was statistically significant (R^2^ = 0.692, adjusted R^2^ = 0.672, F(HC3) = 34.039, *p* < 0.001). At the mean level of social support, emotional labor was positively associated with depression (B = 0.1243, HC3 SE = 0.0604, t = 2.057, *p* = 0.041, 95% CI [0.005, 0.243]). At the mean level of emotional labor, social support was negatively associated with depression (B = −0.2916, HC3 SE = 0.0400, t = −7.287, *p* < 0.001, 95% CI [−0.371, −0.213]), whereas secondary traumatic stress, included as the alternate occupational stressor, was positively associated with depression (B = 0.3680, HC3 SE = 0.0486, t = 7.573, *p* < 0.001, 95% CI [0.272, 0.464]). The emotional labor × social support interaction was statistically significant (B = −0.0121, HC3 SE = 0.0038, t = −3.167, *p* = 0.002, 95% CI [−0.020, −0.005], ΔR^2^ = 0.022), indicating that the association between emotional labor and depression differed according to the level of social support. The conditional association was statistically significant at low social support (B = 0.262, 95% CI [0.097, 0.428]) and at the mean level of social support (B = 0.124, 95% CI [0.005, 0.243]), but not at high social support (B = −0.014, 95% CI [−0.140, 0.112]).

For suicidal ideation, the overall regression equation was statistically significant (R^2^ = 0.814, adjusted R^2^ = 0.800, F(HC3) = 26.385, *p* < 0.001). At the mean level of social support, emotional labor was not significantly associated with suicidal ideation (B = 0.0086, HC3 SE = 0.0397, t = 0.217, *p* = 0.828, 95% CI [−0.070, 0.087]), whereas depression was positively associated with suicidal ideation (B = 0.2176, HC3 SE = 0.0433, t = 5.021, *p* < 0.001, 95% CI [0.132, 0.303]). At the mean levels of emotional labor and depression, the conditional coefficient for social support was negative but not statistically significant (B = −0.0151, HC3 SE = 0.0316, t = −0.478, *p* = 0.633, 95% CI [−0.077, 0.047]). The emotional labor × social support interaction was not statistically significant (B = −0.0025, HC3 SE = 0.0033, t = −0.755, *p* = 0.451, 95% CI [−0.009, 0.004], ΔR^2^ = 0.002), whereas the depression × social support interaction was statistically significant (B = −0.0159, HC3 SE = 0.0026, t = −6.146, *p* < 0.001, 95% CI [−0.021, −0.011], ΔR^2^ = 0.055). Secondary traumatic stress remained positively associated with suicidal ideation in this model (B = 0.1118, HC3 SE = 0.0295, t = 3.790, *p* < 0.001, 95% CI [0.054, 0.170]). Conditional direct and indirect associations across levels of social support are presented in Section 3.4. 

### 3.4. Conditional Direct and Indirect Associations at Levels of Social Support

Because PROCESS Model 59 allows social support to moderate both the a and b paths, a single linear index of moderated mediation was not used. Instead, conditional direct and indirect associations were evaluated at low (−1 SD), mean, and high (+1 SD) levels of social support. In the secondary traumatic stress model, the conditional direct association between secondary traumatic stress and suicidal ideation was statistically supported at low social support (B = 0.236, HC3 SE = 0.059, *p* < 0.001, 95% CI [0.120, 0.352]) and at the mean level of social support (B = 0.140, HC3 SE = 0.033, *p* < 0.001, 95% CI [0.076, 0.205]), but not at high social support (B = 0.045, HC3 SE = 0.033, *p* = 0.177, 95% CI [−0.020, 0.110]). The conditional association between depression and suicidal ideation showed a similar pattern, remaining statistically supported at low and mean levels of social support but not at the high level. The conditional indirect association of secondary traumatic stress with suicidal ideation through depression also became smaller as social support increased and was statistically supported at low (B = 0.191, 95% bootstrap CI [0.114, 0.279]) and mean social support (B = 0.076, 95% bootstrap CI [0.042, 0.114]), but not at high social support (B = 0.013, 95% bootstrap CI [−0.005, 0.035]).

In the emotional labor model, the conditional direct association between emotional labor and suicidal ideation was not statistically supported at low, mean, or high levels of social support. The conditional association between depression and suicidal ideation was statistically supported at low and mean levels of social support but not at the high level. The conditional indirect association of emotional labor with suicidal ideation through depression was statistically supported at low (B = 0.105, 95% bootstrap CI [0.057, 0.227]) and mean social support (B = 0.027, 95% bootstrap CI [0.005, 0.064]), but not at high social support (B = 0.000, 95% bootstrap CI [−0.010, 0.008]). Detailed conditional estimates are presented in Table 6.

Johnson–Neyman analyses indicated that the conditional association between secondary traumatic stress and suicidal ideation was no longer statistically supported above a centered social support value of 9.614 (raw MSPSS score = 55.586). The corresponding thresholds for the depression–suicidal ideation association were 9.172 (raw score = 55.143) in the secondary traumatic stress model and 7.162 (raw score = 53.134) in the emotional labor model.

Given the substantial concentration of suicidal ideation scores at zero, a Tobit sensitivity analysis was conducted. The interaction coefficients remained negative in direction but were not statistically significant in the Tobit models. In the secondary traumatic stress model, the secondary traumatic stress × social support interaction was B = −0.011 (SE = 0.007, *p* = 0.106), and the depression × social support interaction was B = −0.002 (SE = 0.007, *p* = 0.799). In the emotional labor model, the focal predictor × social support interaction was B = −0.002 (SE = 0.005, *p* = 0.751), and the depression × social support interaction was B = −0.006 (SE = 0.006, *p* = 0.308). These sensitivity findings indicate that the moderation effects involving suicidal ideation that were statistically significant in the primary PROCESS analyses did not remain statistically significant under the Tobit specification and should therefore be interpreted cautiously.

## 4. Discussion

This study examined the associations of secondary traumatic stress and emotional labor with depression and suicidal ideation among emergency dispatch personnel, with particular attention paid to statistical indirect and conditional associations involving social support. In the primary PROCESS Model 59 analyses using the total social support score, secondary traumatic stress was positively associated with both depression and suicidal ideation, and the conditional indirect association between secondary traumatic stress and suicidal ideation through depression was statistically supported at low and mean, but not high, levels of social support. Social support also moderated the associations of secondary traumatic stress with depression and suicidal ideation and the association of depression with suicidal ideation. For emotional labor, the direct association with suicidal ideation was not statistically supported, and its conditional indirect association through depression was supported at low and mean, but not high, levels of social support. All interpretations in this section concern statistical associations observed within a cross-sectional design and should not be interpreted as evidence of temporal ordering or causal mechanisms.

At the mean level of social support, secondary traumatic stress was positively associated with depression (B = 0.395, *p* < 0.001) and suicidal ideation (B = 0.140, *p* < 0.001). Although emergency dispatch personnel are not physically present at emergency scenes, they are repeatedly exposed to callers’ screams, cries, and vivid descriptions of crisis events—an occupational context in which indirect trauma exposure may be associated with adverse mental health indicators. This finding aligns with prior work: Adams et al. characterized emergency call-takers and dispatchers as ‘auditory witnesses’ [10], reporting that repeated telephone-mediated exposure to traumatic events is associated with elevated STS, PTSD, depression, and anxiety—with depression showing the highest prevalence among these outcomes [8]. A scoping review of public safety telecommunicators similarly found higher rates of depression and suicidal ideation than in the general population of the United States and Canada, attributing this largely to repeated auditory exposure to potentially traumatic events [16]. A systematic review of emergency telecommunicators reached comparable conclusions, linking auditory trauma exposure among non-field-deployed personnel to depression, STS, PTSD, and suicidal ideation [14,15]. However, because this study did not include a comparison group of field-deployed personnel or assess direct trauma exposure, the findings cannot establish that secondary traumatic stress represents a distinct risk profile relative to field-based trauma exposure.

Depression showed a strong positive association with suicidal ideation. In the secondary traumatic stress model, at the mean level of social support, depression was positively associated with suicidal ideation (B = 0.193, *p* < 0.001), while secondary traumatic stress also showed a positive conditional direct association with suicidal ideation (B = 0.140, *p* < 0.001). The conditional indirect association of secondary traumatic stress with suicidal ideation through depression was statistically supported at low and mean, but not high, levels of social support. Consistent with Hobfoll’s conservation of resources theory [24], this pattern is compatible with the possibility that reduced psychological resources may be associated with greater depressive symptoms in the context of indirect trauma exposure. In the present cross-sectional data, however, the observed indirect association reflects statistical covariation among secondary traumatic stress, depression, and suicidal ideation and does not establish that resource depletion precedes depression or that depression causally transmits the association to suicidal ideation. The strong correlation between depression and suicidal ideation (r = 0.797) also warrants cautious interpretation. Although the two measures assess conceptually distinct constructs, their substantial shared variance may reflect the close relationship between depressive symptoms and suicidal ideation, as well as common-method variance arising from concurrent self-report assessment. This conceptual proximity should be considered when interpreting the magnitude of the observed associations and the statistical indirect effect involving depression. This mirrors findings among Korean firefighters, where PTSD was linked to suicidal ideation indirectly through depression rather than directly [25], and among first responders abroad, where the direct PTSD–suicidal ideation association was non-significant once depression was included as a mediator [26]. A study of rescue workers similarly reported a serial pathway from trauma exposure to posttraumatic stress symptoms (PTSS) to suicidal ideation via depression [27]. Descriptively, the mean CES-D score was close to the screening threshold of 16, and 42.7% of participants scored at or above this threshold; however, these screening-based findings should not be interpreted as the prevalence of clinically diagnosed depression. Collectively, these findings support further evaluation of approaches incorporating depression screening, ongoing monitoring of individuals with elevated psychological symptoms, and timely referral to treatment and counseling among emergency dispatch personnel.

Emotional labor showed a more limited pattern of adjusted associations. It was positively associated with depression at the mean level of social support, and the emotional labor × social support interaction was statistically significant; however, the conditional direct association between emotional labor and suicidal ideation was not statistically supported at low, mean, or high levels of social support. The comparatively limited adjusted associations of emotional labor may partly reflect shared variance with secondary traumatic stress (r = 0.552), although diagnostic indices did not indicate problematic multicollinearity. In addition, because the K-ELS total score combines conceptually distinct dimensions, use of the composite score may have obscured domain-specific associations. Accordingly, the present findings suggest that associations involving emotional labor may vary according to the level of social support, while future studies should examine whether patterns also differ across individual K-ELS domains.

In the primary Model 59 analyses, social support was associated with the strength of several relationships involving secondary traumatic stress and depression. In particular, the positive association between secondary traumatic stress and depression was weaker at higher levels of social support, and this conditional pattern was statistically supported across low, mean, and high levels of social support. This finding is consistent with the stress-buffering hypothesis of social support [30] and with previous studies of emergency dispatch personnel and firefighters reporting inverse associations of social support with trauma-related symptoms and depression [31,32,33]. Given the cross-sectional design, however, these findings should be interpreted as conditional associations rather than evidence that social support causally protects against the psychological consequences of secondary traumatic stress.

In the primary Model 59 analysis with emotional labor as the focal predictor, the association between emotional labor and depression differed according to the level of social support. The association was statistically supported at low and mean levels of social support but not at the high level. This pattern is consistent with the stress-buffering framework, although the cross-sectional design does not establish that social support causally reduces the psychological consequences of emotional labor. Notably, previous studies of Korean social workers and personal care workers for people with disabilities have reported inconsistent evidence regarding moderation by social support [45,46], suggesting that such conditional associations may vary across occupational contexts, sources of support, measurement approaches, and analytic models.

In the primary Model 59 analyses, social support moderated the association between depression and suicidal ideation in both focal-predictor models. The positive association between depression and suicidal ideation was weaker at higher levels of social support and was not statistically supported at the high level of social support. This pattern is consistent with findings among firefighters abroad, where greater social support was associated with a weaker relationship between job stress and suicidal ideation [34]. Given the cross-sectional design, however, these findings should be interpreted as conditional associations rather than evidence that social support causally buffers the effect of depression on suicidal ideation.

Exploratory group comparisons suggested differences in depression and suicidal ideation across several demographic and occupational characteristics. Although participants aged 30–39 years showed higher depression and suicidal ideation scores than the younger and older groups, the present study did not assess the career-stage, family, or organizational factors that might explain this pattern. These findings should be interpreted cautiously because some subgroup sizes were small and substantially unequal, which may have produced unstable estimates and differences in statistical power across comparisons. In addition, multiple subgroup comparisons were conducted. Accordingly, these findings are considered descriptive and hypothesis-generating rather than evidence for identifying particular demographic or occupational groups as being at elevated risk. Replication in larger and more balanced samples is needed before these subgroup patterns can be interpreted substantively.

The present findings suggest several areas that may warrant consideration in the development and evaluation of occupational mental health strategies for emergency dispatch personnel. First, the observed associations of secondary traumatic stress with depression and suicidal ideation suggest that secondary traumatic stress may be a relevant target for monitoring and further evaluation in this workforce. Second, because depression showed a strong association with suicidal ideation, future mental health programs could evaluate whether approaches incorporating depression screening, timely referral, and follow-up are feasible and beneficial for emergency dispatch personnel. Third, the conditional associations involving social support suggest that relational resources may also be relevant targets for future intervention research. Family-inclusive approaches, peer-support initiatives, and supervisor or manager-based support programs could therefore be evaluated in this occupational context, but their effectiveness cannot be inferred from the present study. Finally, although emotional labor showed limited unique adjusted associations in the primary models, its bivariate associations with depression and suicidal ideation and its conditional association with depression at low and mean levels of social support suggest that organizational approaches addressing emotionally demanding work may merit further study. Importantly, this study did not evaluate any intervention; therefore, these implications should be understood as priorities for future program development and testing rather than as evidence of intervention effectiveness.

This study has several limitations. First, the cross-sectional design precludes conclusions about temporal sequence or causal direction among the study variables. Longitudinal research is needed to examine whether the observed statistical indirect and conditional associations are replicated over time. Second, all principal study variables were assessed using self-report measures collected at the same time, raising the possibility of common-method variance as well as reporting biases related to social desirability or the sensitive nature of depression and suicidal ideation. These factors may have inflated or attenuated some observed associations. Third, participants were recruited voluntarily through an online questionnaire accessed via a QR code. Because the exact number of eligible personnel who were exposed to the recruitment notice during the study period could not be determined, a response rate could not be calculated. Individuals who chose to participate may have differed from nonparticipants in their mental health status, interest in the study topic, or willingness to disclose psychological difficulties; therefore, selection and self-selection bias cannot be excluded. Fourth, although participants were recruited from emergency dispatch centers affiliated with two regional fire headquarters, the sample was limited to a single occupational group within the South Korean fire service. Regional and organizational differences may limit generalizability to emergency dispatch personnel in other regions of South Korea, and differences in dispatch systems and occupational roles may further limit international generalizability. Future studies should recruit broader and more diverse samples across regions, organizations, and dispatch systems. Fifth, this study’s measure of social support focused on family, friend, and significant-other support and did not assess coworker, supervisor, or organizational support, which may be particularly relevant in an occupational setting. The very high internal consistency of the MSPSS in this sample (Cronbach’s α = 0.968) may indicate substantial item overlap or shared response tendencies, which should be considered when interpreting the moderation findings involving social support. Sixth, several measurement-related limitations should be considered. Emotional labor was analyzed using the K-ELS total score, which combines conceptually distinct dimensions and may therefore obscure domain-specific associations. Future studies should examine the K-ELS subdomains separately, including their domain-specific and conditional associations with mental health outcomes. The K-STSS used in this study includes intrusion and avoidance items but does not include the arousal subscale of the original STSS, which may limit assessment of arousal-related aspects of secondary traumatic stress that could be particularly relevant to the sustained alertness required in emergency dispatch work. In addition, although the six-item suicidal ideation measure showed high internal consistency in the present study, further validation in adult occupational populations is warranted. Seventh, suicidal ideation scores showed a substantial concentration at zero and a right-skewed distribution, with 65.4% of participants reporting a score of zero. HC3 standard errors were used to account for heteroskedasticity, and a Tobit sensitivity analysis was conducted to assess the robustness of the findings to the zero-heavy outcome distribution. Although the interaction coefficients remained negative in direction, the moderation effects involving suicidal ideation that were statistically significant in the primary PROCESS analyses did not remain statistically significant under the Tobit specification. Accordingly, the conditional process estimates involving suicidal ideation should be interpreted cautiously, and future studies should consider analytic approaches specifically suited to zero-heavy or censored outcome distributions. In addition, the exploratory subgroup comparisons involved multiple comparisons and should therefore be interpreted as hypothesis-generating rather than confirmatory. Taken together, these limitations indicate that future research should prioritize longitudinal and multisite designs, recruit broader and more representative samples, include occupational sources of social support, examine distinct dimensions of emotional labor, and use measurement and analytic approaches that more comprehensively capture secondary traumatic stress and suicidal ideation in adult occupational populations.

## 5. Conclusions

This study highlights secondary traumatic stress and depression as important correlates of suicidal ideation among emergency dispatch personnel. A conditional indirect association between secondary traumatic stress and suicidal ideation through depression was also observed, and this association became weaker at higher levels of perceived social support. Emotional labor showed a more limited pattern of adjusted associations and was not directly associated with suicidal ideation. These findings suggest that future occupational mental health efforts for emergency dispatch personnel could focus on monitoring secondary traumatic stress and depressive symptoms, providing timely referral to appropriate care, and evaluating strategies to strengthen accessible social support. Because the moderation findings involving suicidal ideation were not robust in the sensitivity analysis, they should be interpreted cautiously. Longitudinal and intervention studies are needed to confirm these patterns over time and to evaluate whether such approaches improve mental health outcomes.

## Figures and Tables

**Table 1:** General characteristics (*N* = 211).

Variable	Category	*n* (%)	M ± SD
Sex	Male	150 (71.1)	
	Female	61 (28.9)	
Age (years)	≤29 years	22 (10.4)	39.37 ± 7.88
	30–39 years	97 (46.0)	
	≥40 years	92 (43.6)	
Religion	No	135 (64.0)	
	Yes	76 (36.0)	
Marital status	Unmarried	55 (26.1)	
	Married	145 (68.7)	
	Divorced/Separated	11 (5.2)	
Rank	Firefighter	26 (12.3)	
	Senior Firefighter	51 (24.2)	
	Fire Sergeant	79 (37.4)	
	Fire Lieutenant	40 (19.0)	
	Fire Captain or above	15 (7.1)	
Duty Type	Call reception	112 (53.1)	
	Emergency situation management	37 (17.5)	
	Dispatch control	17 (8.1)	
	Combined duties	45 (21.3)	
Total career duration (years)	<5	39 (18.5)	11.56 ± 7.45
	5–<10	63 (29.9)	
	10–<15	42 (19.9)	
	≥15	67 (31.8)	
Dispatch center work experience (years)	<2	72 (34.1)	4.09 ± 3.76
	2–<4	54 (25.6)	
	≥4	85 (40.3)	

**Table 2:** Exploratory comparisons of main variables according to general characteristics.

Variable	Category	STS	Emotional Labor	Depression	Suicidal Ideation	Social Support
		t/F (*p*)	t/F (*p*)	t/F (*p*)	t/F (*p*)	t/F (*p*)
Sex	Male/Female	−0.832 (0.407) d = −0.114	1.172 (0.243) d = 0.178	0.259 (0.796) d = 0.039	−0.226 (0.822) d = −0.189	−0.766 (0.445)
Age	≤29 ^a^/30–39 ^b^/≥40 ^c^	4.820 (0.009) η^2^ = 0.044	3.983 (0.020) η^2^ = 0.037	5.933 (0.004) η^2^ = 0.057	10.277 (<0.001) η^2^ = 087	17.837 (<0.001)
	post hoc	a < b,c	a < b,c	a,c < b	a,c < b	b,c < a
Religion	No/Yes	0.201 (0.841) d = 0.026	−2.624 (0.009) d = −0.376	2.745 (0.007) d = 0.355	3.642 (<0.001) d = 0.430	−2.569 (0.011)
Marital status	Unmarried ^a^/Married ^b^/Divorced-Separated ^c^	4.200 (0.027) η^2^ = 0.053	8.169 (<0.001) η^2^ = 0.073	5.365 (0.012) η^2^ = 0.115	9.369 (0.001) η^2^ = 0.186	11.646 <0.001
	post hoc	b < c	a,b < c	b < a	b < a,c	c < a,b
Rank	Firefighter ^a^/Sr.Firefighter ^b^/Sergeant ^c^/Lieutenant ^d^/Captain+ ^e^	0.856 (0.495) η^2^ = 0.014	1.932 (0.113) η^2^ = 0.005	4.840 (0.002) η^2^ = 0.041	7.011 (<0.001) η^2^ = 0.078	2.941 0.026
	post hoc	—	—	e < b,c	d,e < b,c	—
Duty type	Call reception/Emerg. mgmt/Dispatch control/Combined	0.583 (0.628) η^2^ = 0.007	0.507 (0.678) η^2^ = 0.007	0.400 (0.753) η^2^ = 0.006	0.715 (0.544) η^2^ = 0.010	1.479 0.221
Total career duration	<5 ^a^/5–<10 ^b^/10–<15 ^c^/≥15 ^d^	4.421 (0.005) η^2^ = 0.060	0.764 (0.516) η^2^ = 0.010	2.734 (0.048) η^2^ = 0.049	5.013 (0.003) η^2^ = 0.086	3.770 0.013
	post hoc	b,c,d < a	—	d < a	d < a	a,c < b,d
Dispatch center experience	<2 ^a^/2–<4 ^b^/≥4 ^c^	2.689 (0.070) η^2^ = 0.025	3.718 (0.026) η^2^ = 0.035	4.053 (0.019) η^2^ = 0.038	3.531 (0.032) η^2^ = 0.029	1.945 0.148
	post hoc	—	b < c	b < a,c	b < a	

Note. STS = secondary traumatic stress. Effect sizes for STS, emotional labor, depression, and suicidal ideation are reported as Cohen’s d for two-group comparisons and η^2^ for comparisons involving three or more groups. Post hoc comparisons were conducted using the Scheffé or Games–Howell test, as appropriate according to the homogeneity-of-variance assumption; “—” indicates no significant post hoc comparison. Superscript letters (a, b, c, d, and e) denote the group labels used for post hoc comparisons. For example, a,c < b indicates that group b had a significantly higher mean than groups a and c. For the marital-status comparisons of secondary traumatic stress and depression, Welch’s ANOVA was used because the homogeneity-of-variance assumption was violated.

**Table 3:** Descriptive statistics, reliability, and correlations among variables.

Variable	M ± SD	Possible Range	Cronbach’s α	STS	Emotional Labor	Depression	Suicidal Ideation	Social Support
				r(*p*)				
STS	29.36 ± 10.63	13–65	0.953	1				
Emotional Labor	66.29 ± 9.81	24–96	0.891	0.552 (<0.001)	1			
Depression	16.04 ± 8.97	0–60	0.869	0.733 (<0.001)	0.422 (<0.001)	1		
Suicidal Ideation	3.07 ± 5.81	0–36	0.972	0.645 (<0.001)	0.293 (<0.001)	0.797 (<0.001)	1	
Social Support	45.97 ± 11.44	12–60	0.968	−0.555 (<0.001)	−0.289 (<0.001)	−0.663 (<0.001)	−0.658 (<0.001)	1

Note. STS = secondary traumatic stress. Possible range refers to the possible total scale score range. Item-level response ranges were 1–5 for STS and social support, 1–4 for emotional labor, 0–3 for depression, and 0–6 for suicidal ideation.

**Table 4:** Primary PROCESS model 59 results for depression.

Variable	B	HC3 SE	t	*p*	95% CI	ΔR^2^
Panel A. Secondary Traumatic Stress as the Focal Predictor						
Secondary traumatic stress	0.3952	0.0463	8.537	<0.001	[0.304, 0.486]	-
Social support	−0.1951	0.0405	−4.822	<0.001	[−0.275, −0.115]	-
STS × Social support	−0.0196	0.0030	−6.530	<0.001	[−0.026, −0.014]	0.058
Emotional labor	0.0890	0.0521	1.710	0.089	[−0.014, 0.192]	-
Model statistics: F(HC3) = 39.302, *p* < 0.001, R^2^ = 0.729; Adj. R^2^ = 0.711						
Panel B. Emotional Labor as the Focal Predictor						
Emotional labor	0.1243	0.0604	2.057	0.041	[0.005, 0.243]	-
Social support	−0.2916	0.0400	−7.287	<0.001	[−0.371, −0.213]	-
Emotional labor × Social support	−0.0121	0.0038	−3.167	0.002	[−0.020, −0.005]	0.022
Secondary traumatic stress	0.3680	0.0486	7.573	<0.001	[0.272, 0.464]	-
Model statistics: F(HC3) = 34.039, *p* < 0.001, R^2^ = 0.692; Adj. R^2^ = 0.672						

Note. B = unstandardized coefficient; HC3 SE = heteroskedasticity-consistent standard error; CI = confidence interval; ΔR^2^ = change in R^2^ associated with the interaction term. Continuous variables involved in interaction terms were mean-centered. Each model was adjusted for the covariates specified in Section 2.4, and the alternate occupational stressor was additionally included as a covariate.

**Table 5:** Primary PROCESS model 59 results for suicidal ideation.

Variable	B	HC3 SE	t	*p*	95% CI	ΔR^2^
Panel A. Secondary Traumatic Stress as the Focal Predictor						
Secondary traumatic stress	0.1404	0.0327	4.292	<0.001	[0.076, 0.205]	-
Depression	0.1932	0.0447	4.319	<0.001	[0.105, 0.281]	-
Social support	−0.0195	0.0285	−0.685	0.494	[−0.076, 0.037]	-
STS × Social support	−0.0084	0.0030	−2.748	0.007	[−0.014, −0.002]	0.009
Depression × Social support	−0.0101	0.0034	−2.964	0.003	[−0.017, −0.003]	0.011
Emotional labor	−0.0015	0.0259	−0.057	0.955	[−0.053, 0.050]	-
Model statistics: F(HC3) = 28.115, *p* < 0.001, R^2^ = 0.821; Adj. R^2^ = 0.807						
Panel B. Emotional Labor as the Focal Predictor						
Emotional labor	0.0086	0.0397	0.217	0.828	[−0.070, 0.087]	-
Depression	0.2176	0.0433	5.021	<0.001	[0.132, 0.303]	-
Social support	−0.0151	0.0316	−0.478	0.633	[−0.077, 0.047]	-
Emotional labor × Social support	−0.0025	0.0033	−0.755	0.451	[−0.009, 0.004]	0.002
Depression × Social support	−0.0159	0.0026	−6.146	<0.001	[−0.021, −0.011]	0.055
Secondary traumatic stress	0.1118	0.0295	3.790	<0.001	[0.054, 0.170]	-
Model statistics: F(HC3) = 26.385, *p* < 0.001, R^2^ = 0.814; Adj. R^2^ = 0.800						

Note. B = unstandardized coefficient; HC3 SE = heteroskedasticity-consistent standard error; CI = confidence interval; ΔR^2^ = change in R^2^ associated with the corresponding interaction term. Continuous variables involved in interaction terms were mean-centered. Each model was adjusted for the covariates specified in Section 2.4, and the alternate occupational stressor was additionally included as a covariate.

**Table 6:** Conditional Estimates at Low, Mean, and High Levels of Social Support.

Path	Social Support Level	Estimate	SE	t	*p*	95% CI
Panel A. Secondary Traumatic Stress as the Focal Predictor						
STS → Suicidal Ideation	Low (−1 SD)	0.236	0.059	4.007	<0.001	[0.120, 0.352]
	Mean	0.140	0.033	4.292	<0.001	[0.076, 0.205]
	High (+1 SD)	0.045	0.033	1.354	0.177	[−0.020, 0.110]
Depression → Suicidal Ideation	Low (−1 SD)	0.309	0.063	4.876	<0.001	[0.184, 0.434]
	Mean	0.193	0.045	4.319	<0.001	[0.105, 0.281]
	High (+1 SD)	0.077	0.055	1.404	0.162	[−0.031, 0.186]
STS → Depression → Suicidal Ideation	Low (−1 SD)	0.191	0.042	-	-	[0.114, 0.279]
	Mean	0.076	0.018	-	-	[0.042, 0.114]
	High (+1 SD)	0.013	0.010	-	-	[−0.005, 0.035]
Panel B. Emotional Labor as the Focal Predictor						
Emotional Labor → Suicidal Ideation	Low (−1 SD)	0.037	0.073	0.503	0.615	[−0.108, 0.182]
	Mean	0.009	0.040	0.217	0.828	[−0.070, 0.087]
	High (+1 SD)	−0.020	0.024	−0.820	0.413	[−0.067, 0.028]
Depression → Suicidal Ideation	Low (−1 SD)	0.400	0.043	9.190	<0.001	[0.314, 0.485]
	Mean	0.218	0.043	5.021	<0.001	[0.132, 0.303]
	High (+1 SD)	0.036	0.060	0.592	0.555	[−0.083, 0.154]
Emotional Labor → Depression → Suicidal Ideation	Low (−1 SD)	0.105	0.041	-	-	[0.057, 0.227]
	Mean	0.027	0.014	-	-	[0.005, 0.064]
	High (+1 SD)	0.000	0.004	-	-	[−0.010, 0.008]

Note. STS = secondary traumatic stress; SE = standard error; CI = confidence interval. Low, mean, and high social support correspond to centered values of −11.44, 0, and +11.44, respectively, equivalent to raw MSPSS total scores of 34.53, 45.97, and 57.41. For conditional direct associations, SE refers to the HC3 standard error and 95% CIs are based on the HC3 estimator. For conditional indirect associations, SE refers to the bootstrap standard error and 95% bootstrap CIs were based on 5000 resamples. Because PROCESS Model 59 specifies moderation of both the a and b paths, a single linear index of moderated mediation was not reported.

## Data Availability

The datasets generated and/or analyzed during the current study are not publicly available due to privacy and ethical restrictions. Data may, however, be made available from the corresponding author upon reasonable request, subject to approval by the Institutional Review Board.
